# From the first descriptions to recent advances: 115 years of reptile *Plasmodium* research in the Neotropics

**DOI:** 10.1590/0074-02760240217

**Published:** 2025-02-24

**Authors:** Erika Martins Braga, Francisco Carlos Ferreira, Irène Landau

**Affiliations:** 1 Universidade Federal de Minas Gerais, Instituto de Ciências Biológicas, Departamento de Parasitologia, Belo Horizonte, MG, Brasil; 2 Texas A&M University, Department of Entomology, College Station, TX, USA; 3 Texas A&M University, Schubot Center for Avian Health, Department of Veterinary Pathobiology, College Station, TX, USA; 4 Muséum Nation d’Histoire Naturelle, Unité Mixte de Recherche, Molécules de Communication et Adaptation des Microorganismes, Paris, France

**Keywords:** Plasmodium diploglossi, Plasmodium tropidurid, Haemosporida, reptiles, taxonomy

## Abstract

Haemosporida research started in the 19th century with the description of *Plasmodium* and other related parasites infecting mammals and birds. Here, we highlight the pioneering contributions of Henrique Aragão and Arthur Neiva in describing the first two *Plasmodium* species in lizards from the New World, *Plasmodium diploglossi* and *Plasmodium tropiduri*, published in the first printed issue of Memórias do Instituto Oswaldo Cruz in April 1909. We use these discoveries as a background to explore some historical and taxonomic aspects of *Plasmodium* species infecting reptiles, with a particular emphasis on the advancements made over the past 115 years in the Neotropics. Our review underscores the complexities and persistent challenges in the taxonomic classification of reptile haemosporidians and discusses some scientific advances in the field that improved our understanding of the biology and evolution of these parasites.

In the late 19th century, the growing interest in parasitology to reveal etiological agents of diseases led to significant biomedical discoveries that invigorated global science. During this period, research on blood parasites in wildlife gained momentum, largely due to the meticulous work of the Ukrainian physiologist and protozoologist Vassily Danilewsky. In 1885, just five years after Charles Louis Alphonse Laveran discovered that a single-celled parasite (*Plasmodium*) was responsible for causing malaria in humans, Danilewsky identified intraerythrocytic parasites in the blood of amphibians, birds, and reptiles. He provided detailed descriptions of the morphology of various forms observed in fresh blood, paving the way for investigating blood parasites in different world regions. The relentless search for parasites in hosts other than humans guided pioneering work in Brazil, led by Adolpho Lutz, to study blood parasites infecting wildlife. From 1893 to 1907, Lutz and his team studied nearly 20 bird orders, finding infections in nine of them. These included species ranging from pigeons and sparrows to owls and hawks, which were used to investigate parasite development.^1^

**Historical context of the discovery of the first reptile *Plasmodium* species in the Neotropics**

In the first decade of the 20th century, Brazilian science was experiencing a period of splendor, marked by achievements that would be later celebrated. In 1909, the young physician Carlos Chagas achieved one of the most remarkable feats in the history of medicine and parasitology. He unraveled the intricate aspects of a disease that would later be named after him. In this historic milestone, the causative agent *Trypanosoma cruzi* was discovered and described, along with its vectors, reservoirs, and clinical forms of the disease. These findings were contextualized within the intricate socioeconomic landscape of Brazil, which shaped and permeated the occurrence of Chagas disease. During his expeditions through Minas Gerais between 1907 and 1908, the curious, attentive, and tireless young physician, inspired by the work by Adolpho Lutz, noticed the presence of hemoparasites in the blood of lizards inhabiting a small village on the banks of the Bicudo River between the cities of Corinto and Curvelo.^2^

In this scientific atmosphere of discovering new parasites and the growing interest in the similarities between the *Plasmodium* species causing human malaria and those infecting other vertebrates, Carlos Chagas sent Giemsa-stained slides to two researchers at the Instituto Soroterápico de Manguinhos, Henrique de Beaurepaire Rohan Aragão and Arthur Neiva. These were blood smears obtained from a specimen of the lizard *Tropidurus torquatus*. According to Carlos Chagas, almost all lizards of this species in the Bicudo region were infected by parasites similar to those causing human malaria. Aragão and Neiva attempted, unsuccessfully, to find infections in *T. torquatus* from other regions of Brazil, including Xerém (now in the municipality of Duque de Caxias in the State of Rio de Janeiro). However, the single sample from the infected *T. torquatus* sent by Carlos Chagas allowed the description of *Plasmodium tropiduri*, and its blood stages were described and illustrated for a publication featuring in the first printed issue of Memórias do Instituto Oswaldo Cruz in April 1909.^3^ This parasite was shown to occupy one end of the red blood cell, displacing the host cell nucleus in late developmental stages. The stages described include round trophozoites, meronts with up to 12 nuclei, and round gametocytes that exhibit clear sexual differences and unevenly distributed malarial pigment (Fig. 1).

**Fig. 1:**
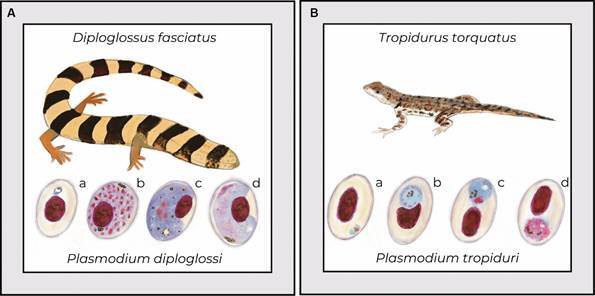
*Plasmodium diploglossi*, *Plasmodium tropiduri* and their respective vertebrate hosts, *Diploglossi fasciatus* and *Tropidurus torquatus*, respectively. Erythrocytic parasite stages: (a) trophozoites, (b) meronts, (c) microgametocyte, (d) macrogametocyte. Illustrations created by Dr Júlia González based on images from Aragão & Neiva, 1909 and Lainson & Shaw, 1969.

In this study, Aragão and Neiva described another species of *Plasmodium* found in two specimens of *Diploglossus fasciatus* from the Xerém region in Rio de Janeiro. These parasites were examined fresh and in Giemsa-stained blood smears, leading to morphological characterization of *Plasmodium diploglossi*. This parasite exhibits characteristics distinct from those reported for *P. tropiduri*, including meronts with up to 40 nuclei and clear deformation of the host cell during erythrocytic development (Fig. 1). During the examination of hanging drop preparations or fresh blood analysis, Aragão and Neiva noted that exflagellation (the formation of gametes derived from microgametocytes) could not be observed, a fact they attributed to the quality of the medium used. One of the lizards exhibiting high parasitemia was kept in the laboratory for various experiments. The authors inoculated parasitized blood into three other lizard species, but blood stages of the parasite were not seen in these passages. Additionally, the same infected lizard was exposed to two mosquito species known to inhabit the same collection area, however, all mosquitoes refused to take blood meals.^3^

Since the description of these two *Plasmodium* species, and of *Plasmodium mabuiae* and *Plasmodium agamae* infecting lizards from Sudan, Africa, by Wenyon in 1908,^4^ the pace of species discovery and description remained slow until the 1960s. It was only in 1966 that the treatise “Malarial Parasites and Other Haemosporidia” was published as a result of the meticulous and detailed work of Percy Cyril Claude Garnham, who compiled at least 30 species and subspecies of *Plasmodium* infecting reptiles.^5^ The increase in the number of descriptions in the following decades is mainly attributed to the persistent efforts of parasitologists studying haemosporidian transmission in reptiles. Notably, Sam R Telford Jr extensively surveyed several lizard populations across countries in Asia, Africa, and the Americas, uncovering significant taxonomic and ecological diversity among lizard *Plasmodium* and related parasites. His systematic studies over three decades are comprehensively documented in a reference book.^6^ Moreover, the work by Ralph Lainson, Jeffrey Shaw and, Irène Landau in the Brazilian Amazon region revealed that the life cycles of malarial parasites and the diversity of their hosts are far more complex than previously thought.^7^ Indeed, Lainson and Shaw, 59 years after the original discovery by Aragão and Neiva, identified the skink *Copeoglossum nigropunctatum* (*Mabuya mabouya* in the original publication), a common lizard, as a new host for *P. diploglossi* and *P. tropiduri* ^8^

***Plasmodium tropiduri*, an example of the complexities of reptile haemosporidian taxonomy**

The taxonomy of *P. tropiduri* generated intense debates in the 1970s^9^ ^,^ ^10^ primarily because parasites of varying sizes that infected multiple lizard genera across Central and South America were “lumped” together into a single species.^11^ Therefore, *P. tropiduri* was classified as a species complex with four subspecies that infect lizards from Costa Rica and Panama to southeastern Brazil:^6^ ^,^ ^11^ *Plasmodium tropiduri tropiduri*,^3^ ^,^ ^11^ *Plasmodium tropiduri caribbense*,^11^ *Plasmodium tropiduri panamense* ^11^ and *Plasmodium tropiduri aquaticum*.^11^ All *P. tropiduri* subspecies exhibit a high degree of similarity in gametocyte shape and size (round to ovoid), with merozoites typically arranged in rosettes or fan-like patterns, the most defining features for these parasites. The primary distinctions among these subspecies lie in the distribution of hemozoin pigments, the formation of cytoplasmic projections in meronts, as well as the relatively large size of meronts and gametocytes compared to the nuclei of uninfected erythrocytes.

These parasites also vary in their host and geographical distribution. It is essential to highlight that reptile taxonomy is also a subject of debate. In this review, we adopted the classification proposed by the Brazilian Society of Herpetology.^12^ While *P. t. tropiduri* infects members of the family Tropiduridae (*Tropidurus* spp.) and Mabuyidae (*C. nigropunctatum*) in southeastern to northern Brazil, Guyana and Venezuela, the other three subspecies have been found infecting anoles lizards (Dactyloidae) in Central America and in the Caribbean (Fig. 2). Interestingly, *Plasmodium t. aquaticum* is associated with riverine habitats, infecting two semiaquatic anoles species in Panama and Costa Rica.^6^

**Fig. 2:**
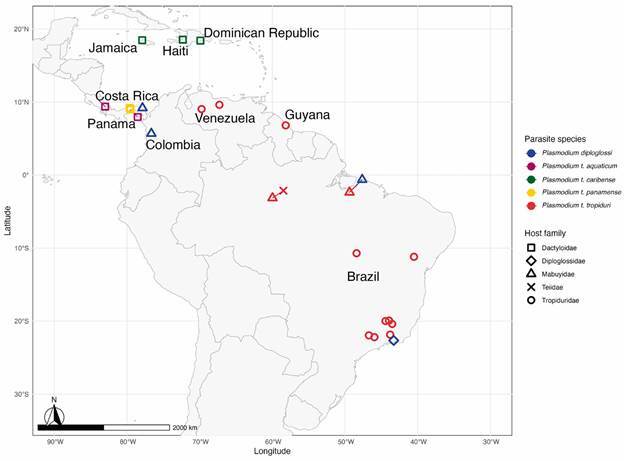
map with the distribution of Plasmodium diploglossi, Plasmodium tropiduri aquaticum, Plasmodium tropiduri caribense, Plasmodium tropiduri panamense, Plasmodium tropiduri tropiduri according to the family of lizard they were reported to infect. Detections of P. t. tropiduri by Silva & Rodrigues^26^ in São Paulo State, Brazil, were consolidated into a single point in the map. The city Quibdo, Colombia, was used as a reference to map the location referred to as “western Colombia” in the description of P. diploglossi in Ayala.^28^

In a study of *Kentropyx calcarata* (family Teiidae) specimens from Pará, northern Brazil, Lainson et al.^13^ found individuals infected with two parasite species. One was newly described as *Plasmodium kentropyxi*, characterized by large, elongated gametocytes and laterally positioned meronts that produce 30-40 merozoites. The second parasite, although having similarities to *P. tropiduri*, such as small, round gametocytes and polar or lateral meronts producing 4-14 merozoites, was designated as *P. tropiduri*-like.^13^ The authors recognized the taxonomic confusion surrounding *P. tropiduri* and chose a tentative classification pending more precise methods to differentiate morphologically similar parasites infecting different host genera. Even after evaluating the ultrastructure of the *P. tropiduri*-like parasite, this classification remained provisional despite the authors’ suggestion that this likely represented a species distinct from *P. tropiduri*.^14^ This context highlights the complexity and ongoing challenges in the classification of reptile hemosporidians, emphasizing the need for continuous revisions and the integration of new criteria and methods to resolve taxonomic issues.

**Integrative approach to unveiling the taxonomy of reptile *Plasmodium***

It is essential to adopt a more integrative approach to better elucidate the diversity and evolution of *Plasmodium* species infecting reptiles. Relying only on traditional morphological and biological studies often limits our understanding of the complexity of these parasites. Morphological characterization of haemosporidians depends on the visualization and measurement of a few two-dimensional structures under light microscopy in most cases, limiting the number of taxonomically valid characters used to classify species.^15^ Therefore, molecular information is particularly useful for distinguishing morphologically similar species^16^ ^,^ ^17^ and serves as a powerful tool for clarifying the classification, taxonomy, population structure, and evolutionary relationships of haemosporidians.^15^ ^,^ ^17^ However, this approach has not yet become a standard in reptile parasite studies.

To our knowledge, polymerase chain reaction (PCR) was employed for the first time to identify *Plasmodium* infections in lizards from California, USA. The authors targeted *Plasmodium mexicanum* using a nested PCR amplification of the 18S small subunit rRNA gene.^18^ Two years later, molecular information linked to a Caribbean reptile *Plasmodium* was established by amplifying and sequencing a fragment of the cytochrome b (*cytb*) gene.^19^ This work exemplifies the use of molecular data to refine the taxonomy of haemosporidians, particularly *Plasmodium azurophilum*, which was initially characterized by its development in both erythrocytes and leucocytes for asexual reproduction and gametogenesis, respectively. Phylogenetic and morphometric analyses combined, however, discerned these two forms as distinct, independently evolving parasite lineages.^19^ Such a critical finding prompted the reclassification of the parasite infecting leucocytes as *Plasmodium leucocytica*,^6^ providing a deeper understanding of its evolutionary biology.

Despite the broad geographical distribution and host range of *P. tropiduri* (Fig. 2) and the extensive application of molecular techniques in parasitology, it took 112 years to assign this parasite species to genetic data. In 2021, our group published the almost complete mitochondrial genome of *P. t. tropiduri* (5420 bp out 5896 bp) infecting *T. torquatus* in southeastern Brazil.^20^ The molecular data we obtained allowed the identification of Brazilian lizard parasites previously detected only through *cytb* sequencing as *P. tropiduri*. Indeed, genetic sequences obtained from *Tropidurus hispidus* and *Hemidactylus mabouia* in the Ceará State^21^ and from *Strobilurus torquatus* in Paraíba State^22^ were identical and almost identical (99.6% similarity at the *cytb* level) to *P. tropiduri*, extending the known geographic distribution of this parasite in Brazil. Describing the genetic diversity of *P. tropiduri* infecting various host species across diverse locations and habitats is essential to delimit parasite species within this complex. This could uncover significant intra-species parasite diversity, given that their hosts inhabit continental and island tropical forests across South and Central America (Fig. 2). For instance, sequencing a *cytb* fragment of *P. kentropyxi* infecting two lizard species, one in the Brazilian Atlantic rainforest and another in the Colombian Amazon rainforest, revealed a 2% genetic difference between these parasite populations.^22^ ^,^ ^23^

Understanding the development of *Plasmodium* parasites within their hosts is crucial for advancing our knowledge of the taxonomy and biology of these parasites. This aspect remains underexplored despite its importance. In 1971, Scorza^24^ described *P. tropiduri* sexual and asexual forms infecting both erythrocytes and thrombocytes from *T. hispidus*. Through experimental infections and electron microscopy, Scorza^24^ hypothesized that parasites infecting thrombocytes and erythrocytes would either constitute different species or that the ability of infecting thrombocytes is a phenotypical trait of *P. t. tropiduri.* The presence of *P. tropiduri* in thrombocytes was also recorded in Brazil.^25^ ^,^ ^26^ In one of these studies,^25^ the analysis of stained blood smears revealed thrombocyte hypertrophy and nuclear displacement, along with evidence of polyparasitism by sexual and asexual stages of the parasite.

Our recent work builds upon these previous findings by incorporating molecular and phylogenetic analyses of parasites infecting *T. torquatus* in Brazil.^20^ We focused on a population of *T. torquatus* harboring haemosporidian infections in erythrocytic and non-erythrocytic cells, and sequencing the mitochondrial genomes of these parasites revealed that different forms constitute divergent genetic lineages. Therefore, the parasite infecting non-erythrocytic cells was characterized as *Plasmodium ouropretensis.* ^20^ This parasite clustered together with *P. leucocytica* ^19^ providing phylogenetic support for the species delimitation proposed. Additionally, these two parasites grouped within the main avian/reptile *Plasmodium* clade, supporting the hypothesis that haemosporidians undergoing merogony in non-erythrocytic cells belong to the family Plasmodiidae.^27^ Characterizing additional haemosporidians infecting non-erythrocytic cells of reptiles in the Neotropics and elsewhere is critical to determine whether this phenotypical trait appeared only once or multiple times in the evolutionary history of this parasite group. This information will likely lead to the redescription of the genus *Plasmodium* to include parasites that infect non-erythrocytic blood cells. Thus, using an integrative approach that combine morphological, biological and, phylogenetic analyses may provide valuable insights into the taxonomy and evolution of Haemosporida.

Since the description of *P. diploglossi* by Aragão & Neiva, this parasite has been found infecting six *C. nigropunctatum* (Mabuyidae) that were sampled in Belém, northern Brazil.^8^ The type host of *P. diploglossi*, *D. fasciatus* (Diploglossidae), is a semi-burrowing species that is difficult to sample. This cryptic behavior was posited as a contributing factor to the delay in its redescription detailed by Lainson and Shaw.^8^ After this redescription, *P. diploglossi* infecting *C. nigropunctatum* were recorded in Colombia^28^ and in Panama.^29^ However, the lack of recent detections of *P. diploglossi*, despite its ability to infect lizards from two distinct families, including the widely distributed *C. nigropunctatum*,^30^ highlights the absence of comprehensive studies on reptile haemosporidians.

Knowledge gaps and future directions 

Although *Plasmodium* parasites are well-studied in mammals and birds, there are still gaps in understanding the taxonomy, diversity, specificity, virulence, and development of these pathogens infecting reptile hosts. Morphological and morphometric analyses reveal remarkable diversity and variability among reptile *Plasmodium.* The high diversity of these parasites in terms of genera and species was suggested to be influenced by reptiles’ limited mobility and isolated habitats, as well as their ancient evolutionary history.^6^ This host group shows significant taxonomic and ecological diversity, with over 12,000 species occupying various habitats and environmental niches,^31^ providing ample opportunities to assess rates of parallel speciation among reptiles and their haemospopridian parasites. Therefore, broad-scale studies applying molecular methods to unveil the diversity of reptile *Plasmodium* and other Haemosporida are essential to address these topics.

Globally, 102 species and subspecies of *Plasmodium* have been identified in reptiles,^6^ ^,^ ^20^ but only 19 of these (18.6%) have genetic data available in public databases.^20^ ^,^ ^23^ In contrast, of the 55 described species of *Plasmodium* infecting birds,^32^ at least 26 species (43.2%) have been linked to genetic sequences.^33^ In the Americas, only 11 species of *Plasmodium* species infecting seven lizard species from four families have corresponding genetic lineages (see Table). Additionally, 49 unique genetic lineages of *Plasmodium*, including those not linked to characterized morphospecies, have been found in reptiles in this region (Table). As a comparison, 753 unique lineages have been identified in birds throughout the Americas.^33^ This discrepancy emphasizes a significant gap in haemosporidian research on reptile hosts and underscores the importance of incorporating molecular tools into the study of these parasites.

**TABLE:** *Plasmodium* genetic lineages at the cytochrome b gene (*cytb*) level detected in reptiles in the Americas

*Plasmodium* species/genetic lineage	Vertebrate host species	Locality	GenBank accession number	Total number of genetic lineages^*a*^	References
*Plasmodium tropiduri*	*Tropidurus torquatus*	Brazil	MW491387	2	^20^
*Plasmodium ouropretensis*	*Tropidurus torquatus*	Brazil	MW491389	2	^20^
*Plasmodium kentropyxi*	*Kentropyx calcarata*	Brazil	MN540144	1	^22^
*Plasmodium* sp. KENCAL02-06	*Kentropyx calcarata*	Brazil	MN540145	5	^22^
*Plasmodium* sp. STRTOR01	*Strobilurus torquatus*	Brazil	MN540150	1	^22^
*Plasmodium* sp. TEC4983	*Tropidurus hispidus*	Brazil	MK033605	1	^21^
*Plasmodium* sp. TEC4774	*Hemidactylus mabouia*	Brazil	MK033603	1	^21^
*Plasmodium* sp. TEC6560	*Ameiva ameiva*	Brazil	MK033604	1	^21^
*Plasmodium* sp.	*Ameiva ameiva*	Brazil	AY099047	1	^46^
*Plasmodium carmelinoi*	*Ameiva ameiva*	Colombia	MF177709	1	^23^
*Plasmodium kentropyxi*	*Cnemidophorus gramivagus*	Colombia	MF177708	1	^23^
*Plasmodium* sp. GU010	*Plica plica*	Colombia	MF177707	1	^23^
*Plasmodium* sp. EB256	*Thecadactylus rapicauda*	Colombia	ON161138	1	^47^
*Plasmodium fairchildi*	*Norops cupreus*	Costa Rica	AY099056	1	^46^
*Plasmodium azurophilum*	*Anolis* spp.	The Caribbean	AY099055; JN187894	8	^19^ ^,^ ^48^
*Plasmidium leucocytica*	*Anolis* spp.	The Caribbean	AY099058; JN187938	6	^19^ ^,^ ^48^
*Plasmodium floridense*	*Anolis oculatus*	Dominica	AY099059	1	^46^
*Plasmodium floridense*	*Anolis spp./Norops* spp.	Central and North America	JN187899	9	^48^ ^,^ ^49^
*Plasmodium hispaniolae*	*Anolis* spp.	Hispaniola	JN187914	3	^49^
*Plasmodium chiricahuae*	*Sceloporus jarrovi*	United States	AY099061	1	^46^
*Plasmodium mexicanum*	*Sceloporus occidentalis*	United States	AY099060	1	^46^

*a*: number of unique genetic lineages associated with a *Plasmodium* parasite according to host species and location.

The *cytb* gene has become a widely used marker in studies reporting reptile haemosporidian diversity in the Neotropics and globally. This use of a genetic “barcode” enables comparisons of parasites across various biomes and geographic regions, facilitating extensive investigations into the molecular diversity and phylogeny of these parasites. Thus, expanding the use of *cytb* and other markers is essential for better understanding the evolutionary relationships among reptile haemosporidians.^34^ It is important to note that current PCR protocols, which target the conserved *cytb* region of the mitochondrial genone, often underestimate mixed haemosporidian infections in vertebrate hosts.^34^ ^,^ ^35^ ^,^ ^36^ Therefore, it is essential to employ cloning or next-generation sequencing (NGS) to fully characterize mixed infections with different species from the *Plasmodium* genus that infect reptiles.^15^ ^,^ ^34^

Lizards are abundant in specific habitats, and some species are easy to observe, capture, and maintain in the laboratory. This facilitates experimental infections with reptile *Plasmodium*, such as sub-inoculating infected blood into non-infected hosts. These experiments can offer valuable insights into parasite biology and its effects on its hosts.^7^ Although Aragão & Neiva^3^ were the first to attempt infecting lizards with *Plasmodium* parasites, experimental models in lizard malaria were established only in the 1940’s with *P. mexicanum.* ^37^ Using *Sceloporus occidentalis* as the vertebrate host, this has become the primary reptile-haemosporidian system utilized in experimental infections in the Americas.^7^ ^,^ ^38^ ^,^ ^39^ Therefore, there are many opportunities for experimental studies involving other reptiles and *Plasmodium* species, with *Tropiduri* spp. and *P. tropiduri* as relevant candidates given their common association in multiple regions within the Neotropics (Fig. 2). These studies are critical to understand parasite dynamics, and could be associated with vector studies, another major gap in reptile haemosporidian research.

Ayala and Lee^40^ discovered that the sandfly species *Lutzomyia vexator* and *Lutzomyia stewartia* in northern California could potentially transmit lizard malaria (*P. mexicanum*), marking the first identification of a non-mosquito arthropod as a putative *Plasmodium* vector. Later laboratory studies confirmed that these sandflies can transmit *P. mexicanum.* ^41^ ^,^ ^42^ Only the *P. mexicanum* - *L. vexator/L. stewarti* model is still used in a few laboratories.^43^ Other researchers have conducted systematic surveys to identify vectors of various common lizard malaria species, but their efforts have yielded limited success.^7^ In Brazil, Nelson Cordeiro also investigated the possibility of *P. tropiduri* transmission through phlebotomine sandflies. *Lutzomyia quinquefer* was abundantly found in the regions where hosts infected with *P. tropiduri* were captured; however, these insects did not show natural infections based on observations of their salivary glands and midguts. Since they could not be maintained in the laboratory, blood-feeding experiments could not be conducted to confirm the vector competence of these invertebrates.^25^

In the same work above,^25^ *Culex quinquefasciatus* and *Aedes fluviatilis* were allowed to feed on lizards infected with *P. tropiduri*. However, no parasite stages, such as oocysts or sporozoites, developed in any of the mosquitoes examined. Twelve years later, Klein et al.^44^ incriminated *Culex* mosquitoes as vectors of lizard *Plasmodium* for the first time. *Culex erraticus* that fed on lizards infected with *Plasmodium floridense* developed mature oocysts in their midguts by nine days post feeding, and sporozoites were observed in the salivary glands after 11 days. Infective mosquitoes successfully transmitted *P. floridense* to uninfected lizards under laboratory conditions. The same study also demonstrated that *P. floridense* did not develop to sporozoite stage in *Culex territans* or *L. vexator*. Future research should prioritize identifying and confirming potential vectors of reptile *Plasmodium*, particularly in the Neotropics, where current vector species remain unconfirmed. Large-scale PCR-based studies targeting blood-feeding Diptera in areas with known transmission of reptile haemosporidians should be a starting point for identifying insect species worth targeting for further investigation. Even though establishing insect colonies in the laboratory remains a significant challenge, experimental confirmation of vector competence is crucial and remains largely unaddressed in the Neotropics and globally.^45^

Concluding remarks

Since the original description of *P. tropiduri* and *P. diploglossi* infecting Brazilian lizards by Araújo & Neiva in 1909, important advances have improved the taxonomy and biology of *Plasmodium* species infecting reptiles. However, substantial gaps remain in our knowledge of their life cycle, vectors, virulence, diversity, and other critical aspects of their biology. The relatively unexplored diversity of reptile haemosporidians provides a unique opportunity to enhance our understanding of parasite taxonomy, evolution, ecology. It also allows us to explore the broader implications of host-parasite interactions for the entire Haemosporida group. To close these knowledge gaps, it is essential to adopt an integrative approach that combines morphological, biological, and molecular data into a cohesive framework.
